# Room Temperature Fabrication of Macroporous Lignin Membranes for the Scalable Production of Black Silicon

**DOI:** 10.1021/acs.biomac.2c00228

**Published:** 2022-05-04

**Authors:** Nadezda Prochukhan, Stephen A. O’Brien, Arantxa Davó-Quiñonero, Anna Trubetskaya, Eoin Cotter, Andrew Selkirk, Ramsankar Senthamaraikannan, Manuel Ruether, David McCloskey, Michael A. Morris

**Affiliations:** † School of Chemistry, Trinity College Dublin, Dublin 2, Ireland; ‡ Centre for Research on Adaptive Nanostructures and Nanodevices (CRANN) and Advanced Materials and Bioengineering Research (AMBER) Research Centres, Trinity College Dublin, Dublin 2, Ireland; § BiOrbic, Bioeconomy SFI Research Centre, University College Dublin, Dublin 4, Ireland; ∥ School of Physics, Trinity College Dublin, Dublin 2, Ireland; ⊥ Department of Bioproducts and Biosystems, School of Chemical Engineering, Aalto University, Espoo 00076, Finland

## Abstract

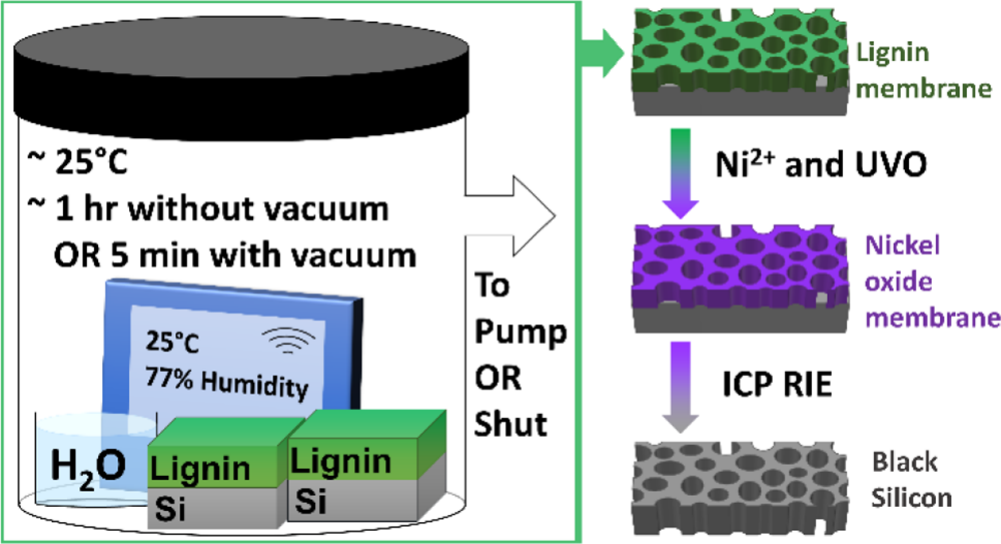

Rising global demand for biodegradable materials and green sources of energy has brought attention to lignin. Herein, we report a method for manufacturing standalone lignin membranes without additives for the first time to date. We demonstrate a scalable method for macroporous (∼100 to 200 nm pores) lignin membrane production using four different organosolv lignin materials under a humid environment (>50% relative humidity) at ambient temperatures (∼20 °C). A range of different thicknesses is reported with densely porous films observed to form if the membrane thickness is below 100 nm. The fabricated membranes were readily used as a template for Ni^2+^ incorporation to produce a nickel oxide membrane after UV/ozone treatment. The resultant mask was etched via an inductively coupled plasma reactive ion etch process, forming a silicon membrane and as a result yielding black silicon (BSi) with a pore depth of >1 μm after 3 min with reflectance <3% in the visible light region. We anticipate that our lignin membrane methodology can be readily applied to various processes ranging from catalysis to sensing and adapted to large-scale manufacturing.

## Introduction

Anthropogenically induced environmental changes have raised awareness about the harmful effects of nonbiodegradable materials. Biopolymer research, in particular, plant-derived materials such as cellulose and lignin research has thus far garnered attraction. Lignin is a biopolymer, only second in abundance to cellulose; nevertheless, it remains largely underutilized and often treated as a byproduct.^1−3^ The main advantages of lignin are biocompatibility, low toxicity, biodegradability, high carbon content, and also carbon neutrality.^4,5^ Lignin is mainly used as a biofuel, an additive for composite materials and the production of added-value chemicals.^4,6,7^ However, lignin membrane technology is currently limited to lignin composite materials, where lignin is not the sole constituent. Therefore, standalone lignin materials are of high commercial and environmental relevance.

The chemical composition of lignin is particularly interesting as it is plentiful with alcohol and methoxy groups, allowing for hydrogen-bonding interactions. Lignin is composed of three polymeric units (monolignols), namely, syringyl, guaiacyl, and *p*-hydroxyphenyl, which in turn consist of sinapyl, coniferyl, and p-coumaryl alcohols, respectively, linked via ether and C–C bonds.^8^ The alcohol groups, in particular, the phenolic hydroxyl groups and carbonyl groups can interact with water and act as active water sorption sites.^9^ The sites of lignin–water interaction can form pore-like features as described by Vu et al. and Wang et al.^10,11^ Therefore, a macroscale porous structure can be obtained, given that the surface area for water exposure is high, that is, the membranes must be below a certain critical thickness.

Lignin is often used as an additive to enhance the tensile strength and rigidity of materials such as membranes.^12,13^ However, to date, there has been no report on standalone lignin membranes without additives. Owing to the highly branched structure of lignin rich with aromatic groups and the large shear modulus (∼GPa range),^14^ lignin-majority membranes tend to be brittle.^15^ Thus, lignin films can be supported on a substrate to overcome this drawback. In the present study, lignin films on silicon (Si) substrates are demonstrated to form porous nanoscale membranes under controlled humidity. Water vapor can act as a nonsolvent-promoting pore formation as described by various researchers.^16−18^ Interactions such as hydrogen bonding and hydrophobic effect result in lignin aggregation, leading to the formations of holes that we call pores.^18^ This process can be called water vapor annealing (WVA) as lignin assembles into porous networks.^19^ Herein, we describe a novel process of sub-micron thickness membrane fabrication from four organosolv lignin materials cast on silicon substrates using only water vapor as a membrane forming agent.

Furthermore, the abundance of alcohol groups and methoxy groups in lignin makes it convenient for metal ion coordination and production of metal oxides. The metal nitrate salts are particularly useful as they dissolve in eco-friendly ethanol or ethanol/water mixtures.^20^ The metal ions then can readily coordinate to the available oxygen lone pairs on lignin upon liquid deposition of metal salt ethanolic solutions. Thus, the lignin structure can be implemented as a template for metal oxide membrane formation. Henceforth, the possible applications could be innumerable, ranging from pattern transfer to production of metal oxide membranes with antimicrobial, sensing, or catalytic properties. The highly uniformly distributed lignin membrane structure yields a nickel oxide membrane upon Ni^2+^ coordination and UV/ozone (UVO) treatment.^21,22^

Additionally, the nickel oxide membrane is an effective inductively coupled plasma (ICP) reactive ion etch (RIE) mask.^23^ Silicon substrates can thus be patterned to create a silicon membrane, which, if the pores are deep enough, forms black silicon (BSi).^24^ The micro- or nanostructured surface on BSi promotes light scattering and absorption as well as reduces reflection losses; thus the surface of BSi appears black.^25^ BSi is therefore highly useful in a wide range of fields spanning from sensing and catalysis to optoelectronic applications, and it is particularly popular in solar cell technology.^26^ Conventional BSi production methods report the use of laser irradiation, metal-assisted chemical etching, wet etching such as HF etching, and RIE used herein among other methods.^27^ RIE processes typically require the use of photoresists or some form of masking material. Therefore, in this work, we demonstrate how a bioavailable and nontoxic lignin material can be used to form the template for pattern transfer without the use of extensive machinery or high costs. Thus, we demonstrate how a so-called byproduct can be used to produce solar cell materials.

The lignin membranes could find other uses in fields such as filtration and separation of large particles such as bacteria,^28^ membrane supports for nanofiltration,^29−31^ and water purification.^32^ Furthermore, lignin itself exhibits antimicrobial properties,^33^ and thus, lignin coatings could be adapted to food packaging.^34^ Similar to nickel ion inclusion, other species with antimicrobial properties such as silver^35^ or zinc ions can be incorporated to produce biocompatible antifouling surfaces. Other potential applications of lignin membranes include energy technology,^36,37^ anti-reflective surfaces,^38^ sensing,^39^ and synthesis of functional materials.^3^

In this work, we demonstrated the formation of lignin membranes of various thicknesses on silicon substrates from four different organosolv lignin materials. A method of nickel oxide membrane production is outlined by liquid deposition of nickel nitrate ethanolic solutions onto lignin membranes with subsequent UVO treatment. A simple ICP RIE method on the nickel oxide mask atop silicon is also used to produce silicon membranes or BSi. We expect that our methodologies of lignin membrane, metal oxide membrane, and BSi fabrication can be further adapted to satisfy the needs of versatile fields from biosensing to photovoltaics.^40^

## Experimental Section

### Materials

The organosolv lignin (**L1**) was supplied by Chemical Point UG (Deisenhofen, Germany) and used without any pretreatment. The softwood lignin (**L2**) and wheat straw lignin (**L3**) were supplied by BOC Sciences and used without pretreatment. The olive stone lignin (**L4**) was sourced from olive stone from the Mediterranean region and prepared as outlined in previous studies^41,42^ via an organosolv process in a steam explosion reactor at 180 °C without an acid catalyst. The pretreated solid was separated from the liquor by vacuum filtration and recovered from the liquor by centrifugation after ethanol removal in a rotary evaporator. The lignin was finally air-dried and stored at room temperature for 18 months.

Silicon <100> p-type wafers and with a native oxide (NO) layer and silicon wafers with a thermal oxide layer (200 nm) were used as substrates. Ni(NO_3_)_3_·6H_2_O (nickel hydrate hexahydrate), ACS reagent, ≥98%), tetrahydrofuran (THF) (inhibitor free), anhydrous ethanol, and acetone (MERCK, Ireland) were high-performance liquid chromatography grade and used as received. Deionized water was used where necessary.

### Lignin Membrane Preparation

Silicon substrates were cleaned in acetone and then in ethanol for 10 min each in an ultrasonic bath. The wafers were then dried with N_2_ gas. Lignin solutions with concentrations from 0.5 to 3%wt were prepared in THF and stirred at 20 °C for 1 week. The substrates were spin-coated with lignin/THF solutions (3000 rpm, 25 s, 5 s ramp). WVA was conducted at room temperature for 5 min to 1 month under ambient atmosphere (20 ± 2 °C, relative humidity ∼50 ± 10%) and in a sealed chamber for 5 to 30 min under 300 ± 50 mbar, 20 ± 2 °C, and a relative humidity of 70 ± 10%.

### Metal Ion Inclusion

Nickel nitrate was dissolved in ethanol to give 0.8 wt % nickel nitrate solution. The nickel nitrate solution was the spin-cast onto the 0.5%wt **L1** lignin samples (3000 rpm, 25 s, 5 s ramp) similar to the methods reported by various researchers.^21,22,43^ UVO treatment was conducted for 3 h via the UV/ozone system (PSD Pro Series Digital UV Ozone System; Novascan Technologies, Inc., USA). Samples were then calcined in a tube furnace at 800 °C for 1 h in order to promote metal densification and remove the residual lignin.

### Pattern Transfer

Pattern transfer was conducted via Oxford Instruments Plasma Technology Plasmalab System100 ICP180 etch tool using nickel oxide as a hard mask. ICP Power of 1200 W and RIE power of 20 W were used, with gas flow rates of 80 and 20 sccm for CHF_3_ and SF_6_, respectively. The chamber pressure was maintained at 16 mTorr. The remaining nickel oxide was removed with 1% oxalic acid (7 h, 20 °C),^44,45^ and the samples were rinsed with DI water and dried with N_2_ gas.

### Characterization

Lignin’s molecular weight was measured by gel permeation chromatography (GPC) according to the previously reported methods^7,8^ and determined to be *M*_n_ = 1115 g mol^–1^ and *M*_w_/*M*_n_ = 7.64 for **L1** lignin; *M*_n_ = 980 g mol^–1^ and *M*_w_/*M*_n_ = 7.16 for **L2** lignin; *M*_n_ = 1484 g mol^–1^ and *M*_w_/*M*_n_ = 10.70 for **L3** lignin; and *M*_n_ = 1050 g mol^–1^ and *M*_w_/*M*_n_ = 4.41 for **L4** lignin.

Ultimate and proximate analysis was performed for all lignin samples. Moisture content was obtained following overnight drying at 105 °C, whereas ash content was determined at 575 °C for the raw samples, following the ASTM standard E1755-01. Ultimate analysis results (CHN) were acquired using a 2400 CHNS/O Series II (PerkinElmer) elemental analyzer, following the procedure in ASTM D5373-02. The oxygen content was calculated by the difference according to previous research.^46^

The thermal stability of Lignin was examined using a PerkinElmer Pyris 1 TGA. Each sample (ca. 3 mg) was loaded in a platinum pan and heated from 30 to 800 °C at the rate of 10 °C/min under nitrogen atmosphere.

A PerkinElmer Pyris Diamond Differential Scanning Calorimeter was used to understand the crystallization behavior of the lignin. The machine was calibrated using indium standards. The samples (∼3 mg) were sealed in aluminum pans and heated from 20 to 250 °C at a rate of 10 °C/min.

Atomic force microscopy (AFM) (Park systems, XE7) was operated in noncontact mode under ambient conditions using a silicon microcantilever probe tip (force constant of 26 N m^–1^) in adaptive scan mode (scan rate of 0.2 to 1.0 Hz, error bound of 1 nm).

Solid-state cross-polarization magic angle spinning (CP/MAS) nuclear magnetic resonance (NMR) spectra of ^13^C were acquired at 100.6 MHz while using a Bruker 400 MHz (9.4 T) Avance HD spectrometer equipped with a 3.2 mm HX-CP/MAS probe. The temperature was stabilized at 20 °C with a flow rate of 600 L/h. ^13^C spectra were acquired under MAS at 20 kHz, if not otherwise specified, using ramped amplitude cross polarization and SWFTPPM decoupling with a ^1^H-decoupling field of about 100 kHz. A 1 ms contact time and a pulse delay of 4 s were used. Chemical shifts were calibrated setting the ^13^C low field signal of adamantane to 38.48 ppm.

Fourier transform infrared (FTIR) spectra were recorded on a PerkinElmer Spectrum 100 equipped with a universal total reflectance (Diamond/KRS-5 sandwich assembly) sampling accessory. The spectra were recorded from 4000 to 400 cm^–1^, with 16 accumulations and a resolution of 4 cm^–1^.

Scanning electron microscopy (SEM, Zeiss Ultra Plus) images were recorded at an accelerating voltage of 2 kV, working distance 4–5 mm, and using a 30 μm aperture. SEM cross sections were obtained from the cleaved samples positioned at a tilt angle of 70°, and the images were recorded using “tilt correction”. The film thicknesses were measured by electron microscopy. Pore size distributions were analyzed from the secondary electron SE2 detector SEM micrographs via ImageJ by applying an FFT bandpass filter to the images, binarizing the images and using the particle analyzer plugin.

Advancing contact angle (CA) measurements were recorded using water, diiodomethane, and glycerol at three different spots on each sample for each liquid. The measurements were conducted on a custom-built system using a 60 Hz camera and a 35-gauge needle (Ø135 μm OD) as described in previous studies.^47,48^ The flow rate was set to 10 nL s^–1^, and the droplet volume was between 80 and 100 nL. CAs were measured on ImageJ using the “drop snake” plugin.^49,50^ Surface energy (SE) was calculated using the advancing CAs via the Lifshitz-van der Waals/acid–base approach.^51^

X-ray photoelectron spectroscopy (XPS) analysis was performed under ultrahigh vacuum conditions (<5 × 10^–10^ mbar) with a non-monochromated source of Al Kα X-rays (1486.6 eV) operating at 200 W (CTX400, PSP Vacuum Technology). The emitted photoelectrons were collected at a take-off angle of 90° from the sample surface and analyzed in a RESOLVE120 spectrometer (PSP Vacuum Technology). XPS spectra were recorded setting the analyzer pass energies constant to 100 and 50 eV for the survey and core scans, respectively. The peak positions of the photoemission lines were corrected to the C 1s transition at a binding energy of 284.8 eV.^52^

### Optical Measurements on BSi

Reflectance measurements in the visible region (400–800 nm) were recorded on BSi samples using a Lambda 650S spectrometer (Perkin Elmer) equipped with an integrating sphere. The spectra resolution of the instrument is 0.17 nm, and the measurements were recorded with a 1 nm step.

Angle-resolved reflectivity measurements were carried out using a custom-built 2θ system, as shown in Figure S12. The system consisted of a Thorlabs halogen lamp weakly focused onto a sample positioned on the inner stage of the rotating 2θ system. The reflected light was collected by means of a collection lens and optical fiber mounted on the outer stage of the system, which rotated at twice the rate of the inner stage. The fiber was connected to an Ocean Optics USB4000 visible range spectrometer with a spectral resolution of 0.2 nm. The measurements were carried out with an angular step of 0.5° and for the wavelength range 400–750 nm (where applicable, values are reported as average value ±1 *s*, where *s* stands for sample standard deviation).

## Results and Discussion

### Membrane Fabrication and Optimization

**Figure 1:**
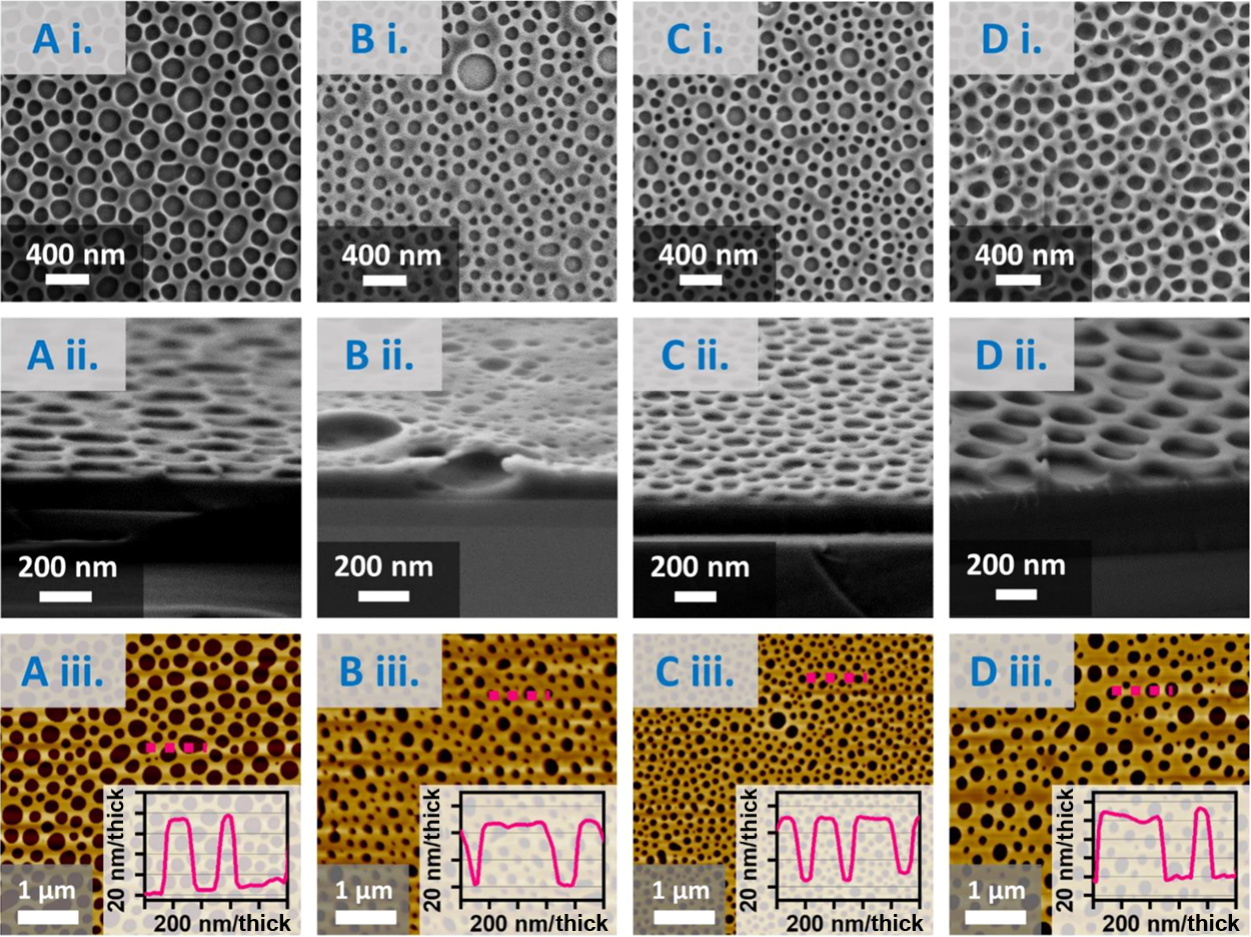
Lignin membranes fabricated in a sealed chamber after 5 min of 0.5 wt % films. (A) Organosolv (**L1**), (B) softwood (**L2**), (C) wheat straw (**L3**), and (D) olive stone (**L4**) lignin membranes. **i.**, **ii.**, and **iii.** represent the top-down SEM, cross-section SEM, and top-down AFM micrographs with line profiles inset, respectively.

In this work, macroporous lignin membranes were produced via spin-coating of four lignin materials onto Si substrates from THF solutions. WVA was applied similarly to our previous work to produce porous films.^22^ Water vapor is chosen over a liquid water treatment in order to prevent the structural collapse. Furthermore, water is readily abundant and low-cost, and it is known to interact with lignin via hydrogen bonding.^53^ Four different lignins, denoted commercial organosolv (**L1**), softwood (**L2**), wheat straw (**L3**), and olive stone (**L4**), were used to fabricate a range of different pore sizes. **L1**, **L2**, and **L3** lignin membranes form readily at 25 ± 1 °C in a WVA assembly^22^ within 5 min to an hour, whereas **L4** membranes required higher humidity, that is, the vacuum chamber assembly (∼300 mbar, ∼20 °C, ∼80% relative humidity, 5 min), which can be due to differences in chemical structure and composition of **L4** lignin. If the laboratory conditions matched the swelling conditions, the films were observed to spontaneously assemble into porous membranes, as shown in Figure 1, after spin-casting, and large-scale SEM images are shown in Figure S1.

Figure 1 portrays the 0.5 wt % lignin membranes derived from **L1**, **L2**, **L3**, and **L4** lignins represented in **A**, **B**, **C**, and **D**, respectively. The thickness of the membranes was estimated from a series of SEM images as 45 ± 8 nm for **L1-**, 43 ± 15 nm for **L2-**, 53 ± 4 nm for **L3-**, and 61 ± 9 nm for **L4**-derived lignin membranes. SEM micrographs and relative thicknesses of membranes produced from 0.8 to 3.0 wt % solutions are shown in Figures S2–S5. The 0.8 wt % membranes display pore sizes similar to 0.5 wt % membranes for all lignins but with a higher thickness. Therefore, we can deduce that membranes with a thickness <100 nm tend to form open pores throughout the structure (Figures S2–S5). The 1 wt % membranes also display a porous network, however, with less open pore space than the 0.5 and 0.8 wt % counterparts for each lignin type. The 1.5 wt % membranes begin to appear less porous as the thickness rises, however, not substantially over 100 nm for all lignins. For higher lignin concentrations, the structures tend toward dense films with less open pore space per volume.

**Figure 2:**
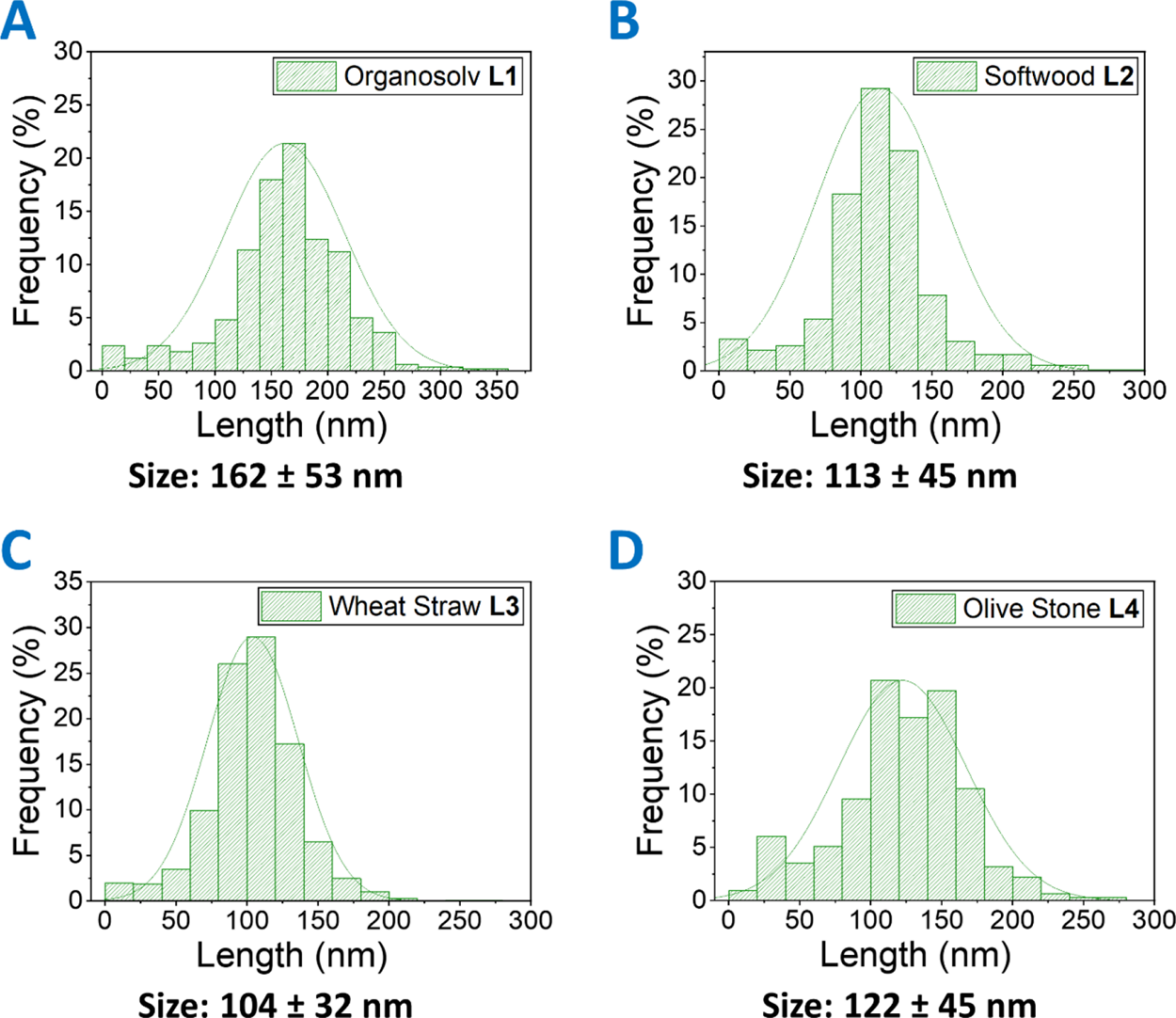
Lignin membrane pore size distributions for 0.5 wt % membranes. (A) Organosolv (**L1**), (B) softwood (**L2**), (C) wheat straw (**L3**), and (D) olive stone (**L4**) lignin membranes.

The pore diameter distributions of the 0.5 wt % membranes are shown in Figure 2–DA for **L1**, **L2**, **L3**, and **L4** lignin membranes, respectively. The membranes display similar pore size distributions, that is, within 100 to 200 nm range. Figure S6 demonstrates that the relationship between molecular weight and pore size is virtually nonexistent; however, the range of molecular weights used is narrow and all lignins are derived and supplied from different sources. The investigation of pore size versus molecular weight is thus a subject of a future study. Furthermore, we investigated how long the obtained porous films remain stable at laboratory ambient conditions.

**Figure 3:**
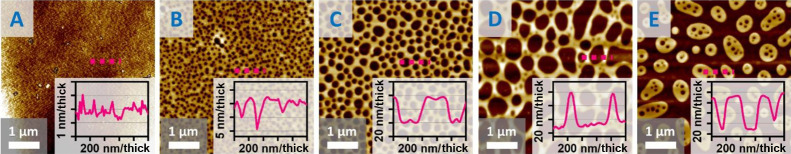
AFM images with line profiles inset of 0.5 wt % **L1** membrane self-assembly stages on a NO Si p-type <100> surface. (A) As-spin-coated film. (B) As-cast film starts to form pores upon WVA within a few minutes. (C) Assembled lignin membrane after 10 min. (D) Pore sizes increase and the film becomes disordered (at 20 °C, 50% relative humidity) after 30 min to a few hours. (E) Film begins to rupture and form lignin islands with smaller pores after ∼24 h at ambient room conditions (20 °C, 50% relative humidity).

To answer the question on how long the membranes remain stable or how long the structure remains uniform, we observed the stability of the membranes over a period of a year on two different substrates: silicon p-type <100> with a NO layer (∼4–6 nm) and silicon p-type <100> with a thermal oxide layer (∼200 nm). The membranes retained the structure on the thermal oxide silicon wafers for 1 to 3 months at ambient conditions (∼20 ± 2 °C, relative humidity ∼50 ± 5%). However, on the NO silicon wafers, the structure undergoes a spontaneous transition to a disordered state forming islands with smaller pores within 24 h to a few days under ambient conditions, as shown for **L1** lignin membranes in Figure 3. Similarly, for SiO_2_ substrates, the film starts to deform, but the process is much slower, that is, several weeks to months. Despite having virtually the same bulk SE after cleaning in acetone and ethanol (45.80 mJ m^–2^ for NO silicon and 47.90 mJ m^–2^ for thermal oxide silicon, as described in Table S1), the substrates are slightly chemically dissimilar. Thermal oxide silicon wafers possess only SiO_2_ at the surface; however, NO is stipulated to contain both SiO and SiO_2_, sometimes described as SiO_*x*_ (*x* < 2) or SiO-SiO_*x*_.^54,55^ We propose that either of the following two processes are involved. Lignin might adhere better to a pure SiO_2_ surface due to the interactions of the lignin groups, or the slightly lower surface energy on the NO surfaces might lead to faster disassembly kinetics causing the lignin to bunch into islands under tension. Lignin is a branched polymer and does not display flow-like motion that is exhibited by straight chain polymers, further explaining film rupturing into islands under prolonged water vapor exposure and due to the surface tension. The **L2**, **L3**, and **L4** membranes follow a similar membrane formation and disassembly stages over time. Interestingly, the lignin films display vastly different bulk SEs (from 26.38 mJ m^–2^ for **L3** to 57.64 mJ m^–2^ for **L1**, as described in Table S1), showing the difference in surface chemistries. Yet all the SE values for lignin membranes are below the water equilibrium surface tension value of 72 mJ m^–2^,^56^ as expected for hydrophobic organosolv lignins. Therefore, despite the difference in SEs, the hydrophobic interactions between water and lignin and the π–π interactions of lignin with itself due to the high abundance of aromatic groups are sufficiently strong to yield similar porous lignin films ubiquitously distributed along the substrate.

The site-specific interactions of lignin with water virtually breaks up the bulk structure and leads to the formation of “pores” due to high surface tension. A more detailed proposed mechanism is as follows: initially, lignins interact strongly with itself mainly by π–π stacking as lignin molecules are flat and highly concentrated in aromatic groups. As water permeates through the film, the lignin molecules aggregate together within the internal surface of the dense film due to hydrophobic effect induced by water and also the lignin π–π interaction with other lignin molecules.^17,18^ This leads to fracturing of the thin layer of the remaining lignin at the outer surface due to a pressure difference, creating the so-called pore. Therefore, a normal phase separation model does not describe the lignin membrane formation well. Under the fluctuations in the atmospheric conditions, the lignin films can alter morphology from porous to dense to ruptured porous lignin islands, as observed in this work. The formation of islands can be akin to formation of spheres in solution by π–π interactions between the aromatic rings within lignin, as described by Xiong et al. and Li et al.^17,18^ To establish the real mechanism of macropore formation (pores >100 nm in diameter), further studies and perhaps novel models of polymer interactions should be designed.

The solid-state CP/MAS ^13^C NMR spectroscopy, FTIR spectroscopy, thermogravimetric analysis (TGA), and differential scanning calorimetry (DSC) characterization of lignin materials can be found in Section S2 and Figures S7–S9.

The four lignin materials used in this study are organosolv lignins with an established general structure.^42^ The main monomers such as syringyl and guaiacyl can be identified from the FTIR and ^13^C CP/MAS NMR spectra, with the assignment included in Figure S7 and Tables S2 and S3. The organosolv lignins used in this paper were extensively structurally characterized in previous studies by various researchers.^57−59^ The FTIR and NMR spectra were, thus, used to verify that the structures of all the organosolv lignins are guaiacyl–syringyl types with predominantly aromatic groups.^60−62^ The **L1** and **L2** lignins were observed to have higher aromaticity than the other two lignins and **L4** lignin was deduced to have the highest purity. In general, the four lignins behave similarly under WVA with an assembly mechanism described above pertaining to all the materials. **L4**, however, required a vacuum chamber for self-assembly, which is explained by the low aromaticity and high purity, that is, lower propensity for lignin π–π interactions and reduced hydrophobic effect during WVA. A more detailed structural analysis is included in the Supporting Information below Tables S2 and S3.

The XPS survey spectra of the lignin membranes are shown in Figure S10 with associated elemental content atop NO silicon wafers. The proximate and ultimate analysis results are displayed in Table S4. The elemental composition for all lignins is similar. The DSC analysis shows that L1, L2, and L3 lignins have a glass transition at approx. 60 to 65 °C, whereas the s lignin displays a glass transition temperature *T*_g_ of ∼41 °C. The glass transition in this case is observed as an endothermic peak as described by Gordobil et al., whereby enthalpy relaxation takes place.^63^ Those workers postulate that lignin kept for large periods of time at conditions close to glass transition exhibits improved chain mobility, which explains the endothermic peak. All the lignins remain stable up to 200 °C, as seen from TGA curves in Figure S8, so there is no thermal degradation during the membrane fabrication process.

### Nickel Oxide Membranes

A well-known metal oxide inclusion methodology can be adapted to the lignin membranes to yield nickel oxide membrane structures. Lignins are rich with polar groups, with lone pairs available for metal coordination such as alcohols; therefore, we anticipated incorporation of the metal into the structure ubiquitously. Nickel nitrate 0.8 wt % ethanolic solution was spun on as in previous studies to fabricate a lignin membrane with coordinated Ni^2+^ ions.^22^

**Figure 4:**
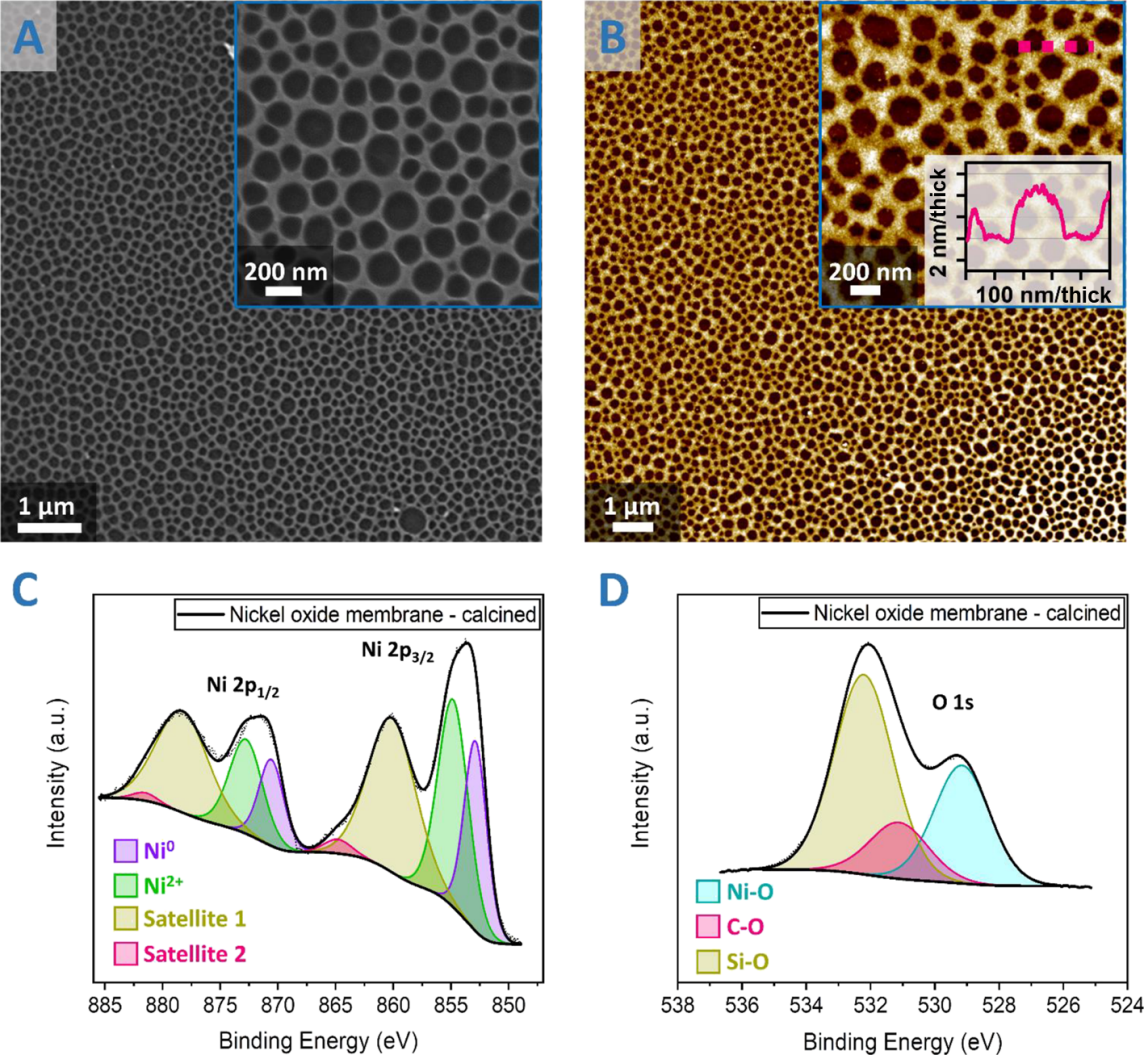
Nickel oxide membrane after calcination produced from the **L1** 0.5 wt % lignin membrane: (A) SEM images and (B) AFM images with a line profiles inset. The XPS high-resolution core scans spectra of (C) Ni 2p and (D) O 1s of nickel oxide membrane on NO silicon after calcination.

To investigate the nickel membranes, we selected **L1** 0.5 wt % membranes due to the lowest cost of the source material. After metal inclusion, a UVO treatment was conducted to remove the lignin material and to produce the nickel oxide. A further calcination step, that is, heating at 800 °C for 1 h was carried out to remove any residual lignin. The nickel oxide membranes show a relatively uniform structure, as seen in Figure 4,BA, with a pore size (144 ± 37 nm) similar to the lignin membrane (162 ± 53 nm). The size variation in pore size after nickel oxide infiltration, UVO treatment, and calcination compared to the lignin polymer membrane is not significant.

The survey spectra of nickel oxide after UVO treatment and after calcination is shown in Figure S11A,B. Prior to calcination, the nickel mask consists of solely NiO [Ni 2p_1/2_ (872.3 eV) and Ni 2p_3/2_ (854.7 eV)], as shown in Figure S11C, as opposed to block copolymer nickel oxide masks after UVO [Ni(II) and Ni(III) oxide].^21^ We propose that lignin-bound Ni^2+^ ions are prevented from further oxidation to Ni^3+^ and thus NiO is the only oxide. The O 1s core spectrum also indicates the presence of only one oxide NiO at 530.0 eV as well as C–O (530.9 eV) and Si–O (532.5 eV) bonds.

The chemical composition of the nickel oxide membrane after calcination was determined to be NiO (Ni^2+^) with a minor amount of nickel metal (Ni^0^) as seen from curve-fitted binding energies.^64^ After UVO, the NiO has a lot of surface defects because of the UVO-lignin removal in the surrounding, as described by the “container” effect.^65^ This generates labile oxygen groups that upon heating at 800 °C leads to a partially reduced state, that is, Ni metal formation. Figure 4C shows that the curve-fitted (Gaussian–Lorentzian) binding energies for nickel oxide are Ni 2p_1/2_ (870.6 eV) and Ni 2p_3/2_ (852.9 eV) assigned to Ni^0^ and Ni 2p_1/2_ (872.8 eV) and Ni 2p_3/2_ (854.8 eV) assigned to Ni^2+^. The O 1s core spectrum shows only C–O (531.1 eV), Si–O (532.2 eV), and Ni–O (529.2 eV) chemical linkages (Figure 4D). We expect that the content of nickel metal in the mask facilitates improved etching depth as compared to just solely NiO masks.

The method of Ni^2+^ coordination to the lignin substrate can also be used to “store” the structure as the nickel ions tether the lignin in place and prevent polymer motion and disassembly. We applied this method to preserve the structures for several months on either substrate without subsequent UVO treatment, as shown in the AFM image in Figure S12A for an **L1** 0.5 wt % sample with incorporated Ni^2+^ ions.

### Silicon Membranes and Optical Applications

**Scheme 1:**
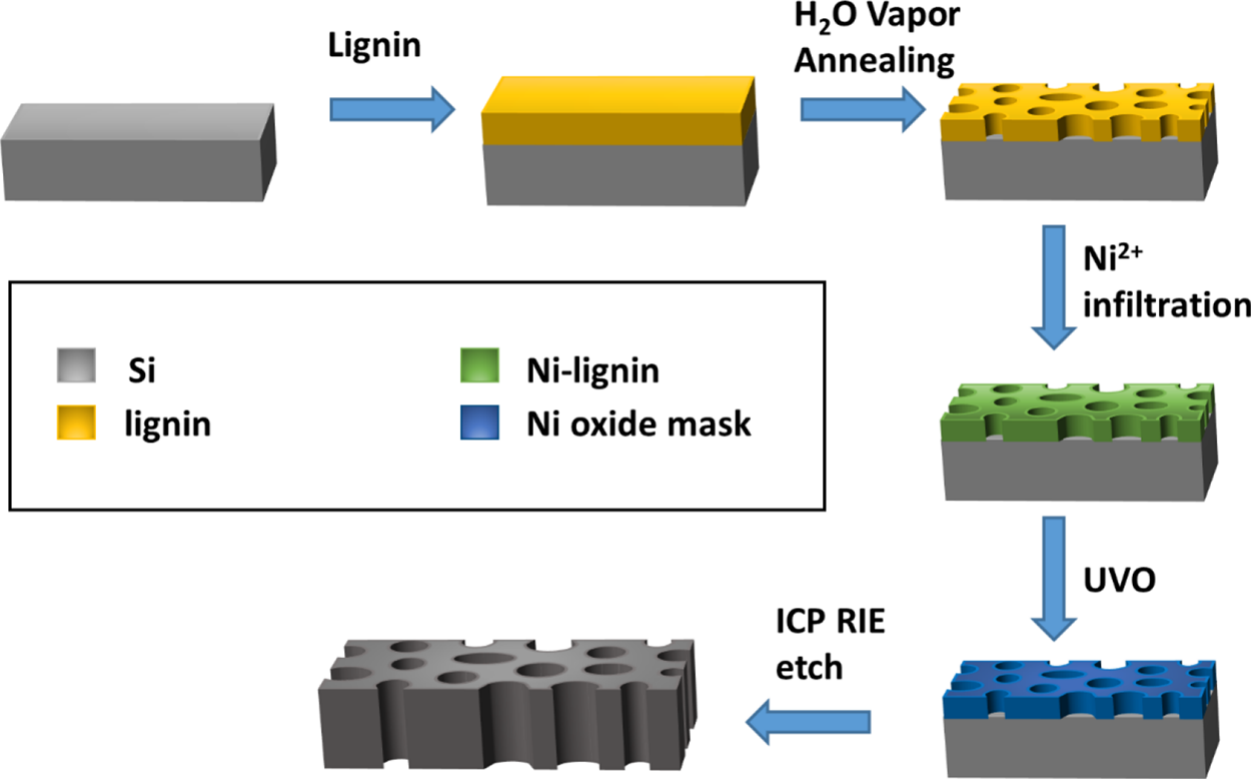
Overall Lignin-Based Silicon Membrane Production Process The lignin is spun from THF solutions ono silicon substrates. Further, the nickel precursor is deposited, and the lignin is removed via UVO. The pattern is transferred onto the silicon substrate via an ICP RIE etch.

Furthermore, pattern transfer was applied to etch the silicon substrates to create an ordered membrane with cylindrical macroscale pores. The full process is described in Scheme 1.

**Figure 5:**
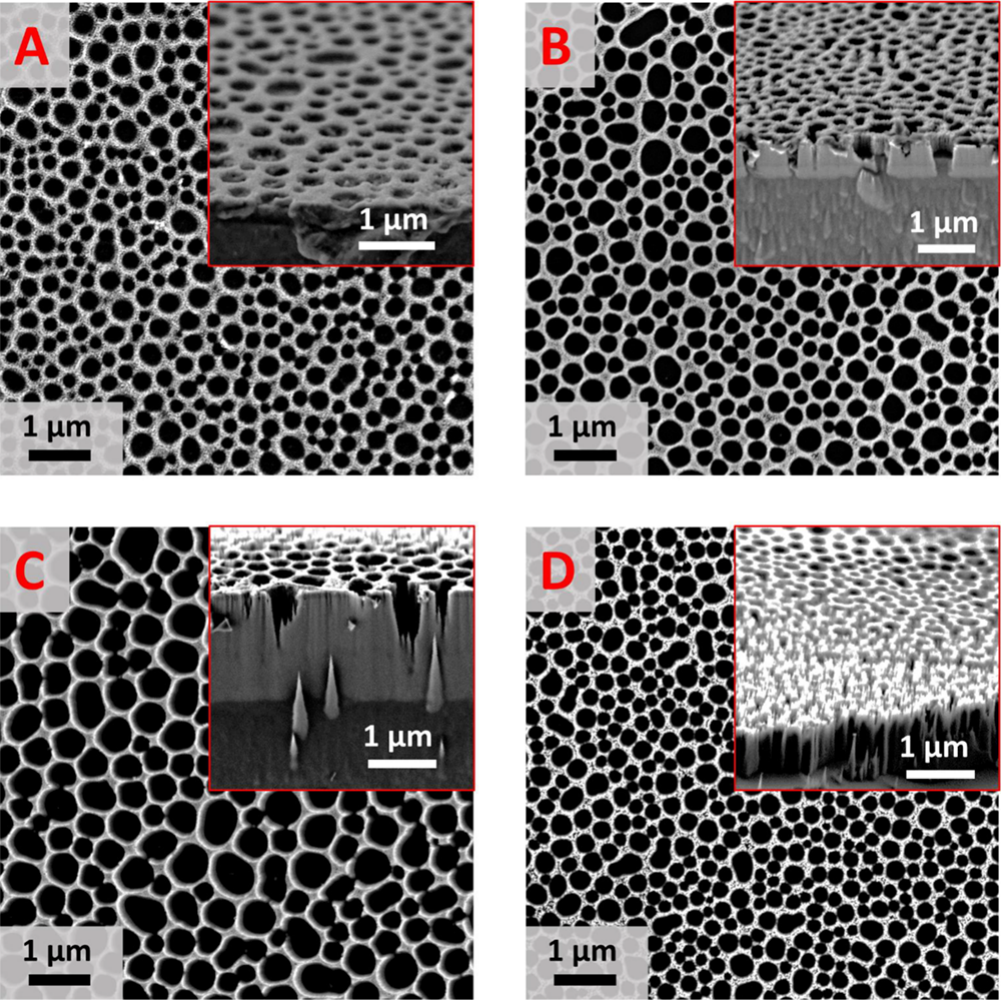
SEM images with cross-section inset silicon membranes produced from **L1** 0.5 wt % lignin membrane nickel oxide template after ICP RIE etch after (A) 1 min, (B) 2 min, (C) 3 min, and (D) 4 min.

The calcined nickel oxide mask was etched for 1 to 4 min, as shown in Figure 5–DA. A 1 min etch produces pore etch depth of 150 ± 25 nm, and a 4 min etch leads to mask degradation with large variations in feature heights. The 2 and 3 min etch yield a pore depth of 406 ± 66 and 1307 ± 201 nm, respectively. However, upon the ICP RIE etch, the pore diameter increases over a 100 nm in size from 144 ± 37 nm (nickel oxide mask) to 256 ± 64 nm (after 1 min etch) (Figure S12B,C), possibly due to etch mask degradation during etching. The pore size distributions after 1 to 4 min etches are practically the same. For the purpose of BSi production with a large etch depth (>1 μm), the size increase is irrelevant, but where the mask pore size needs to be preserved, this aspect should be noted.

**Figure 6:**
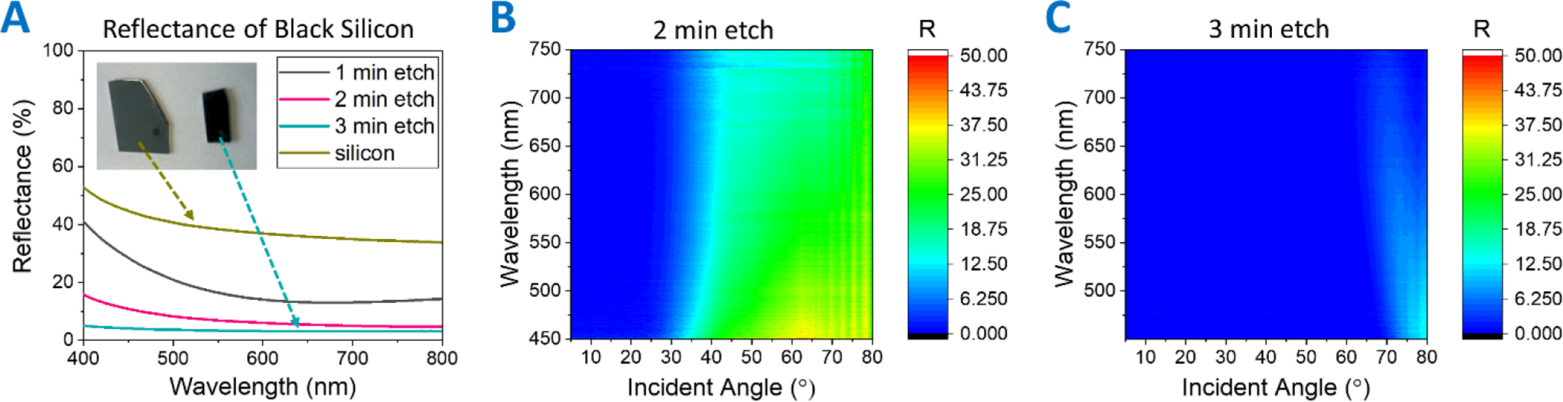
(A) UV–vis–NIR spectra of NO silicon, 1, 2, and 3 min etch samples, (B) angle-resolved visible spectrum of the 2 min etched sample, and (C) angle-resolved visible spectrum of the 3 min etched sample.

Standard and angle-resolved reflectance measurements were recorded to understand the optical behavior of the silicon membranes and to determine the suitability of the process to BSi production. Figure 6A shows the normal reflection spectra of an unetched silicon sample with NO (4–6 nm), compared with samples etched for 1, 2, and 3 min, respectively. The average reflection from the NO sample across the visible range at normal incidence is 38%. This is reduced to 18% for the 1 min etch, 7.1% for 2 min etched sample, and then <3% for the 3 min etched sample. Furthermore, the 3 min etched sample appears black throughout, as shown in the inset photograph in Figure 6A. Thus, the extensive distribution of the etch pattern over a macroscale area coupled with the extremely low reflectance in the visible region make the 3 min etch process suitable for large-area BSi production.

It is essential to look at the angle-resolved reflectance measurements for our process to be practically useful. The angle-dependent reflectivity for the samples was taken using a custom-built 2θ system with the setup shown in Figure S13. The surface plots show the magnitude of unpolarized reflection as a function of angle on the *x*-axis and wavelength on the *y*-axis. In general, we expect to see low reflectivity for a broad range of angles but an increase at higher angles due to Fresnel reflection. The 2 min etch results in lower reflection at normal incidence; however, it still has large reflection at higher angles (Figure 6B). The 3 min etched sample shows a strong reduction in reflection which persists even at the higher angles (Figure 6C).

Previous studies that reported BSi production by ICP RIE methods either used expensive materials such as Pt^66^ or textured the surface to produce pillars of higher reflectance than reported herein.^27^ The coating of the textured silicon substrates with Al_2_O_3_,^67^ NbN,^68^ or other materials coupled with the use of highly energy demanding atomic layer deposition^69^ or other methods improved the reflectance to 3%.^27^ However, our work shows that BSi can be produced with reflectance lower than 3% in the visible region without the use of costly materials, coatings, or machinery. In summary, the 3 min etch sample displays desirable attributes for BSi manufacturing such as low reflection over a broad range of wavelengths and angles and macroscale coverage of the etched structure. Therefore, we recommend our lignin and nickel membrane methodology with the 3 min ICP RIE process as a suitable candidate for solar cell applications.

## Conclusions

We demonstrated a completely novel and highly facile strategy to fabricate large-area macroporous membranes derived from lignin without additives. The four chosen lignin materials yielded pores in the range of 100 to 200 nm, when membrane thickness remained below 100 nm. The process is highly scalable and produces large-area porous films. The described lignin membranes are easy to produce on a large scale with simple equipment and chemicals. We suggest that this structure can be adapted to incorporate various moieties such as metal oxides for optical, antimicrobial, or sensing applications.

Furthermore, we demonstrated nickel oxide membrane production from the lignin templates via a facile method of metal infiltration, UVO treatment, and calcination. These metal oxide structures have potential applications in areas such as sensing or catalysis. These metal oxide templates were then utilized as etch masks for the fabrication of silicon membranes (with pore depths of over 1 um) via a well-established ICP RIE method. The BSi produced displayed low reflectivity (<3% for the 3 min etch sample) in the visible range. We therefore anticipate that the BSi produced by this method can be applied to solar cell technology.

Overall, we believe that this work greatly expands the potential and scope of lignin bulk chemistry and membrane formation processes. This method is comparatively cheaper and more eco-friendly than the existing polymer lithographic techniques as both lignin and water are abundant and ecologically safe. We anticipate that the lignin membranes and methodology described in this work open a new research direction for the development of a variety of photonic and catalytic applications.

## Supporting Information Available

Additional SEM and XPS data, solid-state ^13^C NMR, FTIR, TGA, DSC, and proximate and ultimate analyses of lignins, contact angle and surface energy measurements, additional size distributions and reflectance data, additional AFM, and the schematic of the custom-built 2θ system (PDF)
