# Supplementary material for: Room Temperature Fabrication of Macroporous Lignin Membranes for the Scalable Production of Black Silicon
Source: Biomacromolecules. 2022 May 4;23(6):2512–21. doi: 10.1021/acs.biomac.2c00228 (PMC9198978; doi:10.1021/acs.biomac.2c00228)
Supplement: Supplementary file 1 — bm2c00228_si_001.pdf [file bm2c00228_si_001.pdf]

# Supporting Information

## Room Temperature Fabrication of Macroporous Lignin Membranes for the Scalable Production of Black Silicon.

Nadezda Prochukhan,[a,b,c,*] Stephen A. O'Brien,[b,d] Arantxa Davó-Quiñonero,[a,b] Anna Trubetskaya,[e] Eoin Cotter,[b,d] Andrew Selkirk,[a,b] Ramsankar Senthamaraikannan,[a,b] Manuel Ruether,[a] David McCloskey,[b,d] Michael A. Morris[a,b,c,*]

[a]*School of Chemistry, Trinity College Dublin, Dublin 2, Ireland;*

[b]*Centre for Research on Adaptive Nanostructures and Nanodevices (CRANN) and Advanced Materials and Bioengineering Research (AMBER) Research Centres, Trinity College Dublin, Dublin 2, Ireland;*

[c] *BiOrbic, Bioeconomy SFI Research Centre, University College Dublin, Dublin 4, Ireland;*

[d] *School of Physics, Trinity College Dublin, Dublin 2, Ireland.*

[e] *Department of Bioproducts and Biosystems, School of Chemical Engineering, Aalto University, 00076 Espoo, Finland*

## Section S1 – Lignin membranes of higher thicknesses.

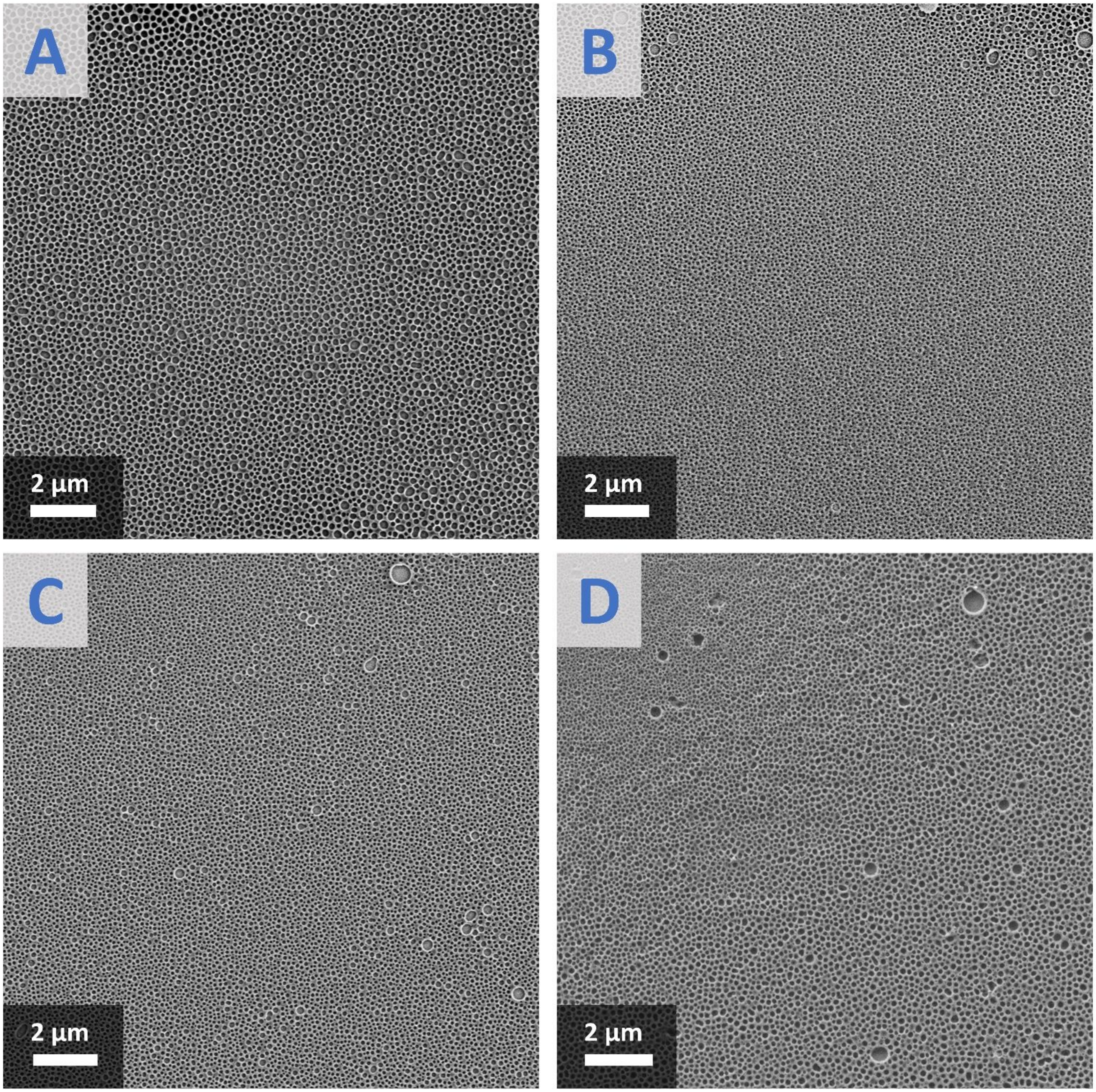


**Figure S1.** Large area SEM micrographs of 0.5 wt% lignin membranes: **A. L1**, **B. L2**, **C. L3** and **D. L4.**

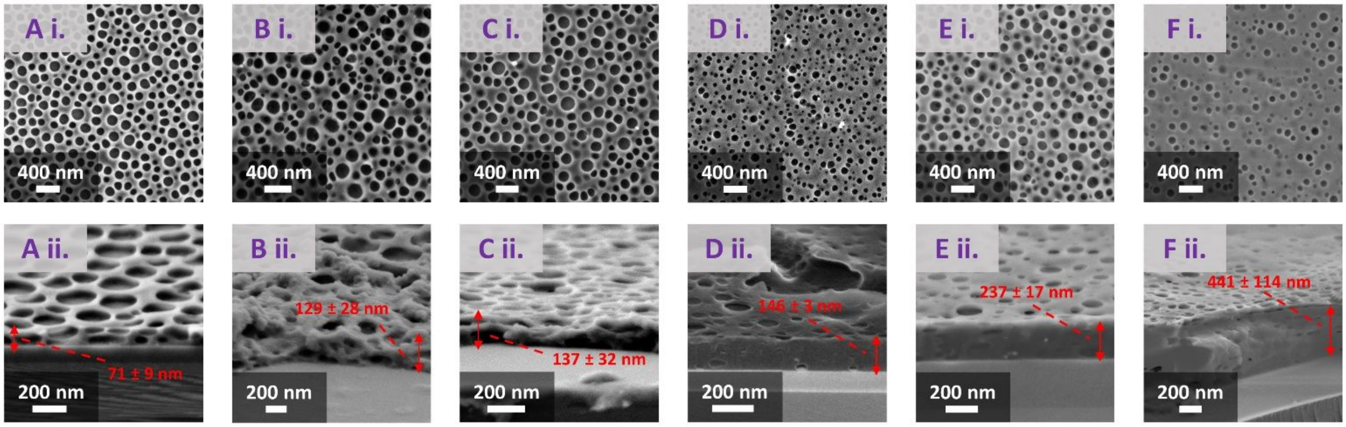


**Figure S2.** SEM micrographs of **L1** lignin membranes where **A** is 0.8 wt%, **B** is 1.0 wt%, **C** is 1.5 wt%, **D** is 2.0 wt%, **E** is 2.5 wt%, **F** is 3 wt% membranes; **i.** represents the top-down SEM and **ii.** represents the cross-section SEM micrographs with associated thicknesses.

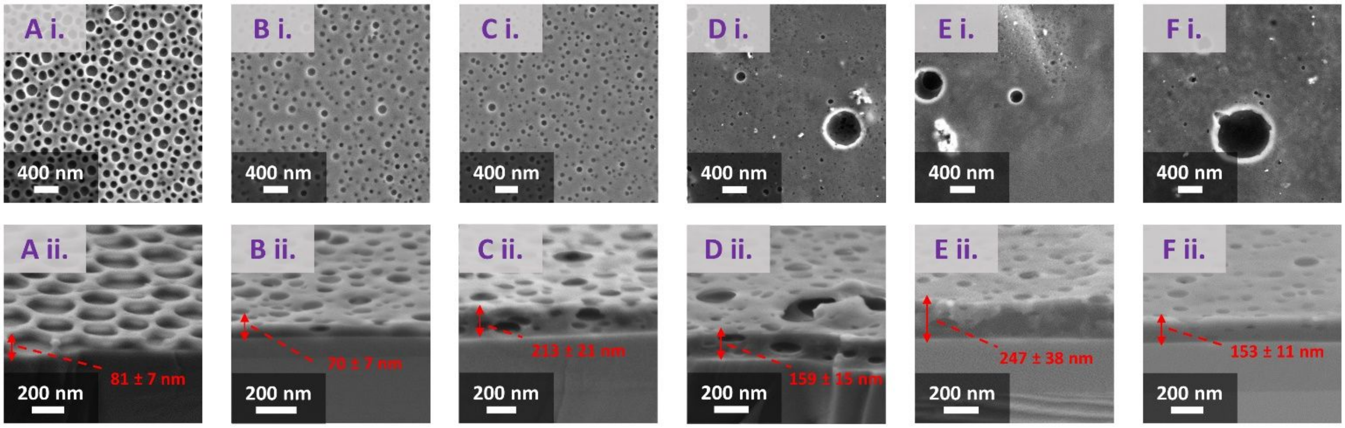


**Figure S3.** SEM micrographs of **L2** lignin membranes where **A** is 0.8 wt%, **B** is 1.0 wt%, **C** is 1.5 wt%, **D** is 2.0 wt%, **E** is 2.5 wt%, **F** is 3 wt% membranes; **i.** represents the top-down SEM and **ii.** represents the cross-section SEM micrographs with associated thicknesses.

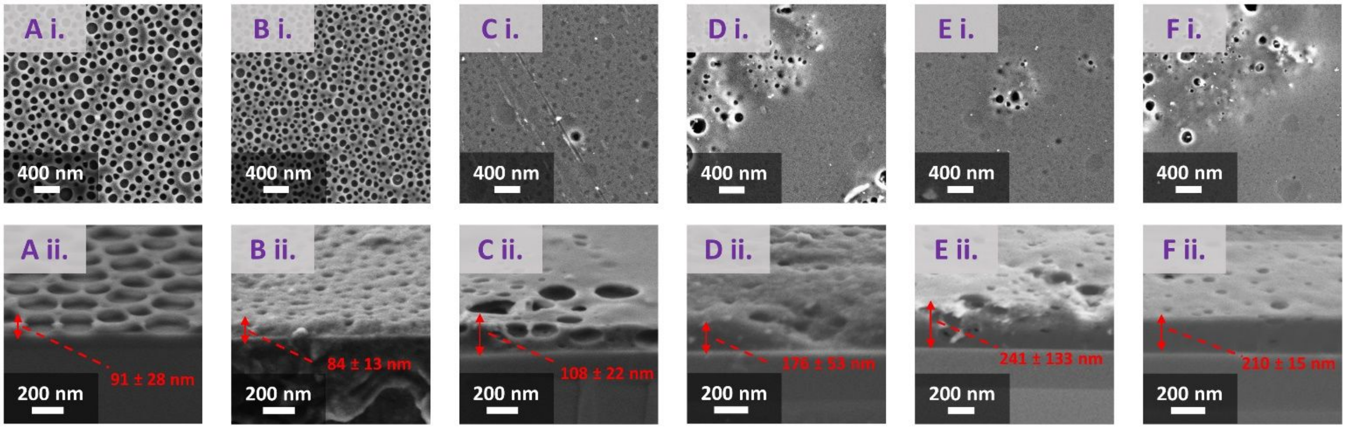


**Figure S4.** SEM micrographs of **L3** lignin membranes where **A** is 0.8 wt%, **B** is 1.0 wt%, **C** is 1.5 wt%,

**D** is 2.0 wt%, **E** is 2.5 wt%, **F** is 3 wt% membranes; **i.** represents the top-down SEM and **ii.** represents the cross-section SEM micrographs with associated thicknesses.

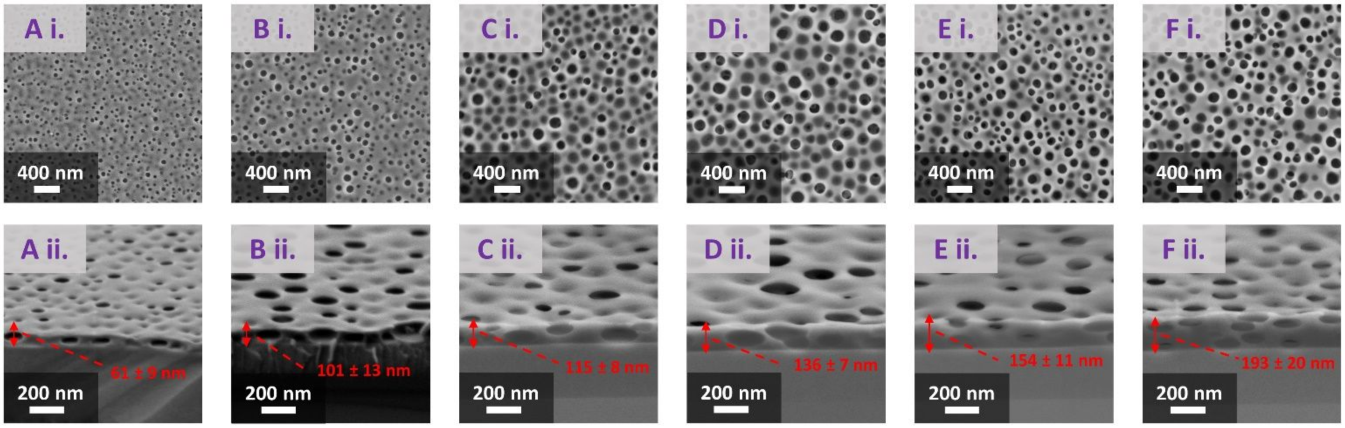


**Figure S5.** SEM micrographs of **L4** lignin membranes where **A** is 0.8 wt%, **B** is 1.0 wt%, **C** is 1.5 wt%, **D** is 2.0 wt%, **E** is 2.5 wt%, **F** is 3 wt% membranes; **i.** represents the top-down SEM and **ii.** represents the cross-section SEM micrographs with associated thicknesses.

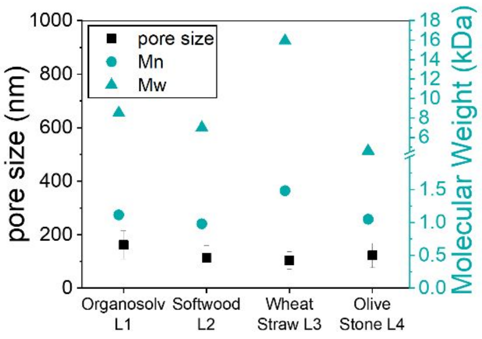


**Figure S6.** The comparison between pore sizes of 0.5 wt% membranes and molecular weight: the number average molecular weight ($M_n$) and weight average molecular weight ($M_w$). There appears to be no observable correlation between pore size and molecular weight.

# Section S2 – Supporting Characterization.

**Table S1.** Contact angle and surface energy measurements of 0.5 wt% lignin membranes and silicon substrates.

| Sample | Contact angle (°) | | | Surface Energy (mJ m$^{-2}$) |
|---|---|---|---|---|
| | Water | Diiodomethane | Glycerol | |
| Organosolv **L1** | 70.94 ± 0.75 | 48.43 ± 0.94 | 89.82 ± 1.69 | 57.64 |
| Softwood **L2** | 85.87 ± 1.31 | 44.63 ± 1.14 | 80.69 ± 1.47 | 39.50 |
| Wheat Straw **L3** | 112.01 ± 1.54 | 70.81 ± 0.61 | 82.64 ± 0.27 | 26.38 |
| Olive Stone **L4** | 74.92 ± 0.32 | 45.28 ± 0.76 | 65.23 ± 0.87 | 40.12 |
| Silicon native oxide | 52.21 ± 1.37 | 58.97 ± 0.35 | 46.53 ± 1.24 | 45.80 |
| Silicon thermal oxide | 54.76 ± 1.20 | 58.88 ± 0.86 | 38.81 ± 1.67 | 47.90 |

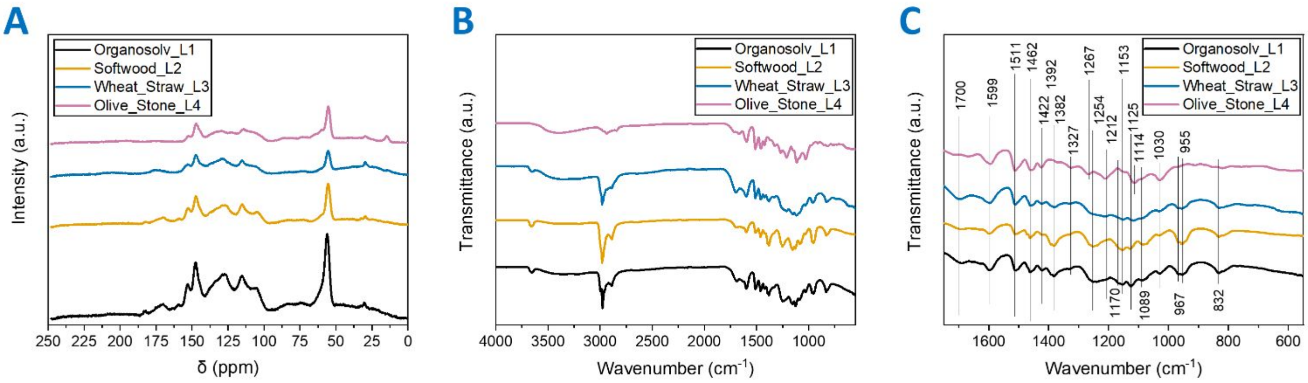


**Figure S7. A.** $^{13}$C CP/MAS NMR, **B.** and **C.** FTIR spectra of **L1**, **L2**, **L3** and **L4** lignins in the 4000 – 550 cm$^{-1}$ and 1750 – 550 cm$^{-1}$ respectively for clearer interpretation.

**Table S2.** The $^{13}$C CP/MAS NMR chemical shifts assignments of the four lignins according to Trubetskaya et al.[1,2] Abbreviations: S, syringyl; G, guaiacyl; ne, in non-etherified arylglycerol b-aryl ethers; e, in etherified arylglycerol b-aryl ethers.

| Chemical shift, ppm | Assignment |
|---|---|
| 170 – 174 | Carbohydrate; –COO–R, $CH_3$–COO– |
| 152 – 154 | Lignin; S3(e), S5(e) |
| 147 – 148 | Lignin; S3(ne), S5(ne), G1(e), G4(e) |
| 133 – 138 | Lignin; S1(e), S4(e), G1(e) |
| 127 – 130 | Lignin; G6 |
| 105 – 116 | Carbohydrates; C1, Lignin; S2, S6, G5, G6 |
| 89 – 92 | C4 in cellulose (cr) |
| 82 – 86 | C4 in cellulose (am) |
| 72 – 75 | C2, C3 in carbohydrates; C5 in cellulose |
| 55 – 57 | Lignin, $OCH_3$ |
| 29 – 40 | $CH_2$ in aliphatics |

**Table S3.** The FTIR vibrational bands assignments of the four lignins according to various researchers.[1,3–6]

| Wavenumber, $cm^{-1}$ | Assignment |
|---|---|
| 3400 | OH stretch |
| 3000 – 2930 | -$CH_2$- stretch |
| 2930 – 2830 | -$CH_3$- stretch |
| 1700 | C=O stretch |
| 1599 | C=C aromatic skeletal vibration |
| 1511 | C=C aromatic skeletal vibration |
| 1462 | C-H asymmetric deformations in $CH_2$ and $CH_3$ groups |
| 1422 | C-H asymmetric deformation in -$OCH_3$ |
| 1400 – 1360 | O-H and C-O of phenol and tertiary alcohol, aliphatic C-H stretch in $CH_3$ |
| 1327 | C-O stretching in syringyl ring breathing |
| 1270 – 1250 | C-O stretching in guaiacyl ring breathing |
| 1212 | C-O stretching in syringyl and guaiacyl ring breathing |
| 1153 | C-H in-plane deformation of guaiacyl ring |
| 1125 | C-H in-plane deformation of syringyl ring |
| 1114 | C-O deformations of secondary alcohols and aliphatic ethers aromatic C-H in-plane deformation (G > S) |
| 1089 | C-O deformation in secondary alcohols and aliphatic esters |
| 1030 | C-OH stretching of the side groups and C–O-C and glycosidic bonds |
| 970 – 950 | =CH out-of-plane deformation (trans) (guaiacyl) |
| 832 | C-H out-of-plane deformation of guaiacyl ring |

The NMR characterization of the lignin samples indicates a presence of residual carbohydrates predominantly in the **L1**, **L2** and **L3** lignins. The **L4** sample shows much lower relative intensity in the region of 170 – 174 pm indicating that the pre-treatment removes most of the impurities. Furthermore,

cellulose is also observed in all the lignin materials. The intensity of cellulose peaks are comparatively low in all the lignin samples indicating low amount of residual impurities in general. The NMR assignments in **Table S2** confirm the presence of both syringyl and guaiacyl units.

The FTIR analysis demonstrates that the lignins **L1**, **L2** and **L3** show guaiacyl ring breathing as a shoulder in the 1250 – 1270 $cm^{-1}$ region whereas the **L4** lignin has a well-defined peak at 1267 $cm^{-1}$ indicating higher purity. Furthermore, the signal at 1327 $cm^{-1}$ responsible for the syringyl ring breathing is better defined for **L4** lignin compared to the other three materials (broad signal) indicating higher purity of **L4**. The C=C presence is confirmed in all the four lignins (1599 $cm^{-1}$ and 1511 $cm^{-1}$) indicating high content of aromaticity.[1] The four lignins display aromatic C-H deformations at 1153 $cm^{-1}$ but **L3** and **L4** lignins have significantly lower signal intensity indicating lower aromatic content than **L2** which has a slightly less intense signal than **L1**. It is possible to deduce that **L1** has the higher aromatic content than **L2**, and **L3** and **L4** show lower content than both. The **L1** lignin displays an additional peak at 1170 $cm^{-1}$ responsible for C=O vibration of esters[1] but **L2** has lower intensity with **L3** and **L4** showing no signal.

Guaiacyl and syringyl is identified in all the four lignin samples from both NMR and FTIR. However, it can be observed that **L4** lignin has less impurities than the other three lignin materials. Therefore, the differences in structure i.e., degree of aromaticity and the content of impurities can explain why L4 lignin requires a vacuum chamber for the self-assembly to occur. This can be due to two factors – either the degree of aromaticity or impurities result in less energy demanding self-assembly onset. The degree of aromaticity improves π-π interactions of lignin with itself and thus enhances the hydrophobic effect of water allowing for easier self-assembly. Furthermore, impurities can act as nucleation sites for pore formation to occur as they break up the lignin aggregated structure and allow for faster self-assembly kinetics.

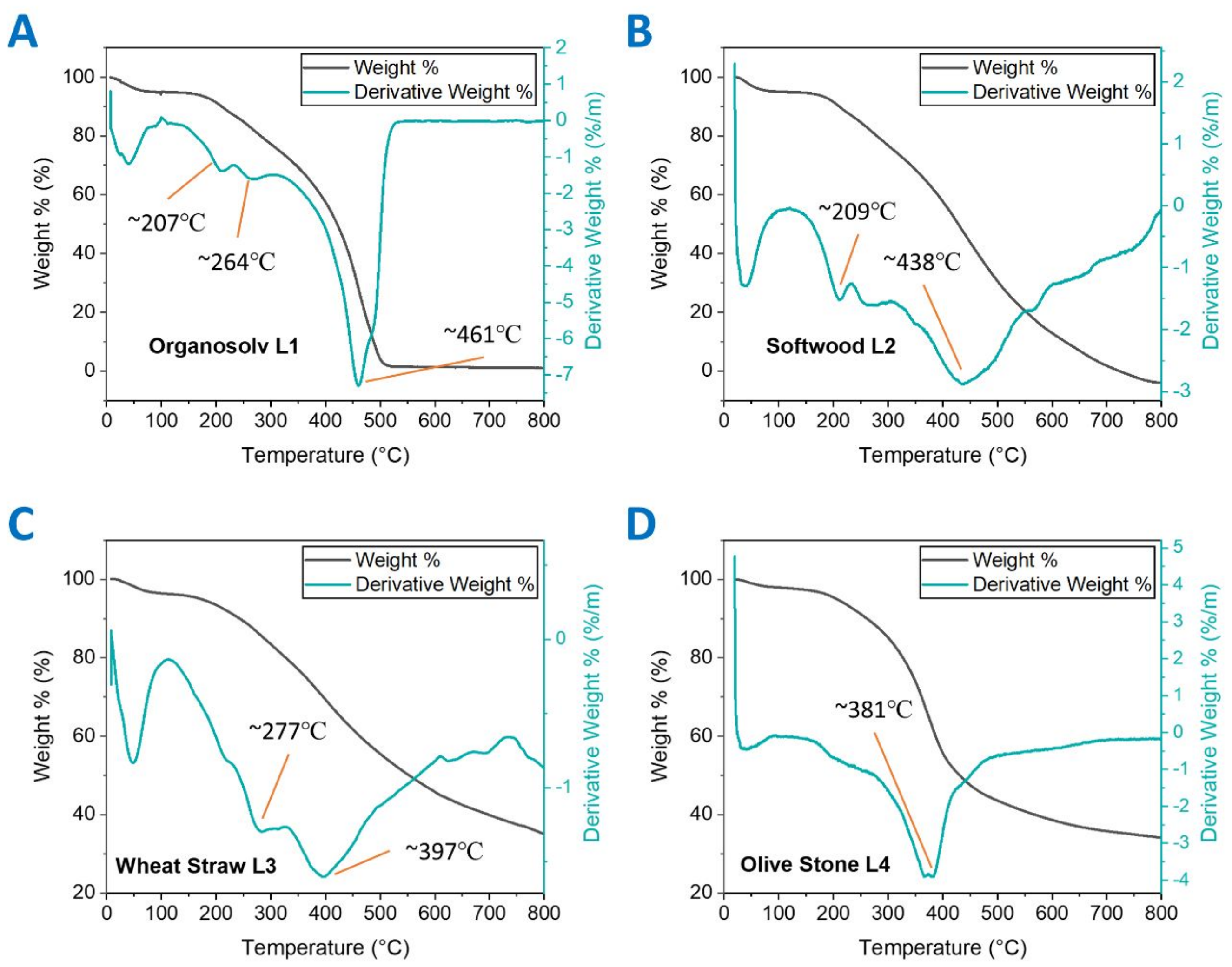


**Figure S8.** TGA data of **A. L1**, **B. L2**, **C. L3** and **D. L4** lignins.

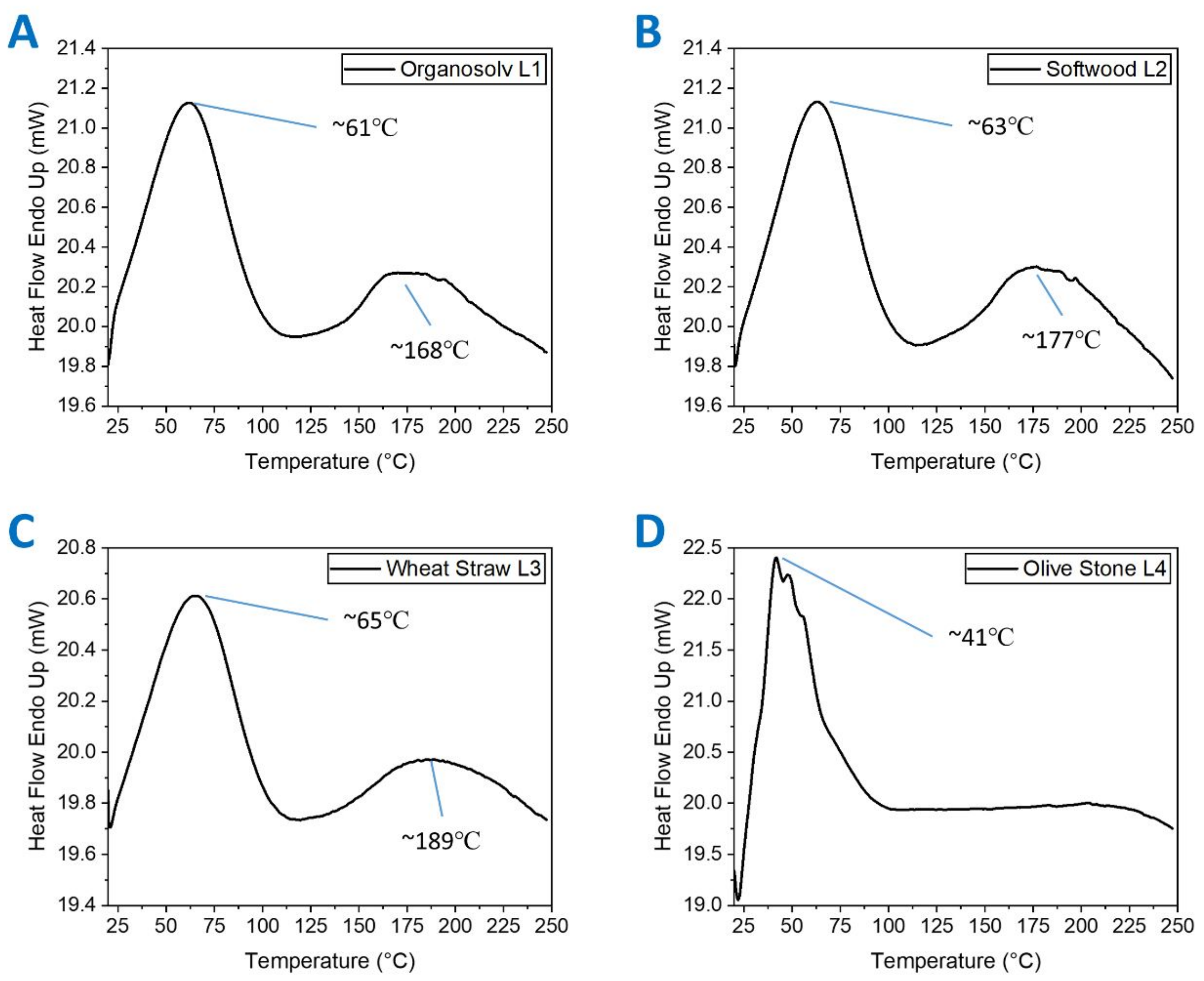


**Figure S9.** DSC data of **A. L1**, **B. L2**, **C. L3** and **D. L4** lignin materials.

**Table S4.** Proximate and ultimate analysis of **L1**, **L2**, **L3** and **L4** lignins.

| | Organosolv L1 | Softwood L2 | Wheat Straw L3 | Olive Stone L4 |
|---|---|---|---|---|
| *Proximate and ultimate analysis (% on dry basis)* | | | | |
| Moisture | 1.5 | 4.1 | 3.8 | 3.7 |
| Ash (550 °C) | 3.8 | 1.5 | 4.3 | 1.6 |
| C | 61.7 | 59.9 | 61.1 | 64.7 |
| H | 5.3 | 5.5 | 5.6 | 5.6 |
| O | 28.3 | 31.9 | 28.2 | 27.6 |
| N | 0.9 | 1.2 | 0.8 | 1.0 |

## Section S3 – Supporting XPS.

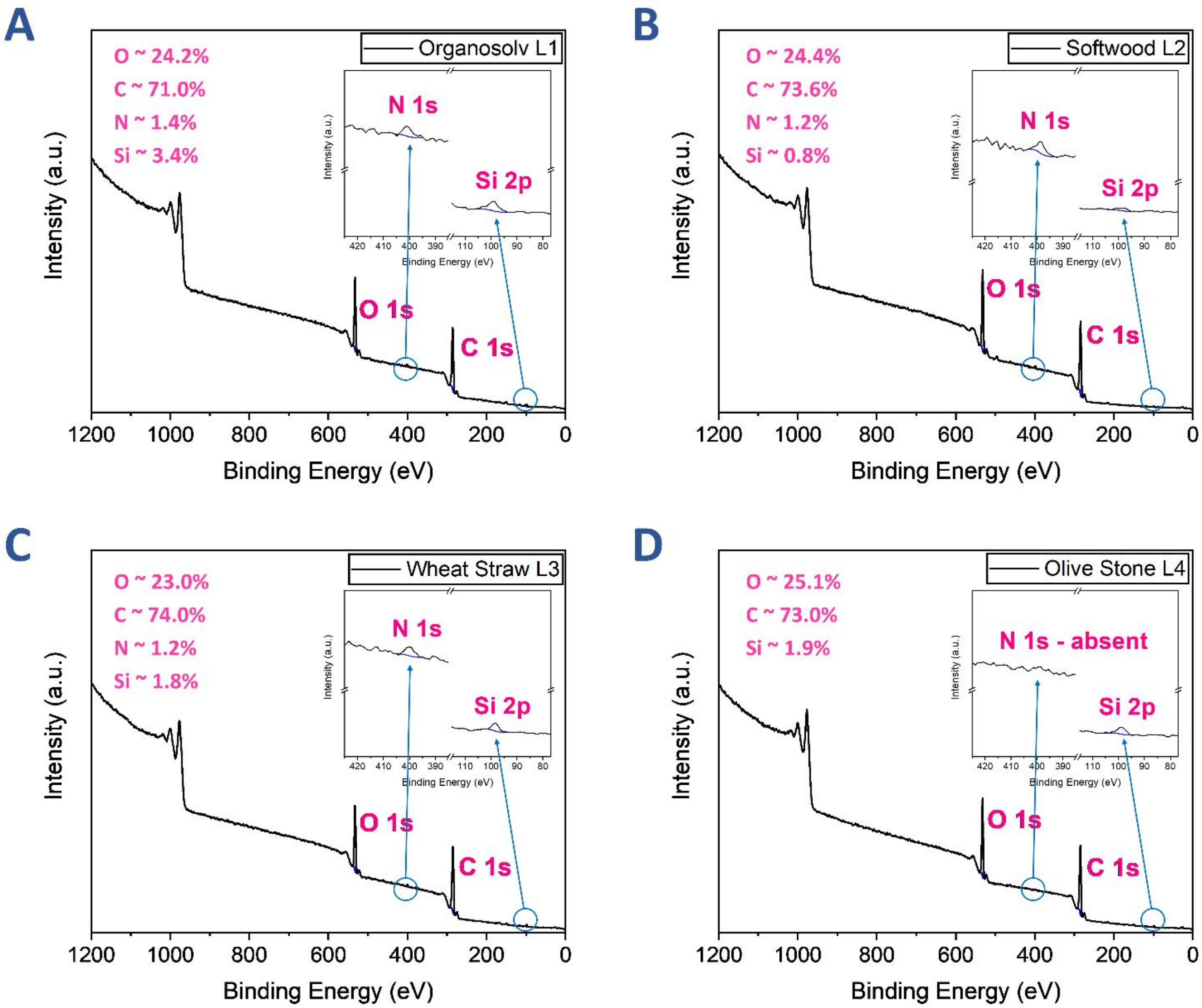


**Figure S10.** Survey XPS spectra and elemental composition of **A. L1**, **B. L2**, **C. L3** and **D. L4** lignin membranes on native oxide silicon wafers.

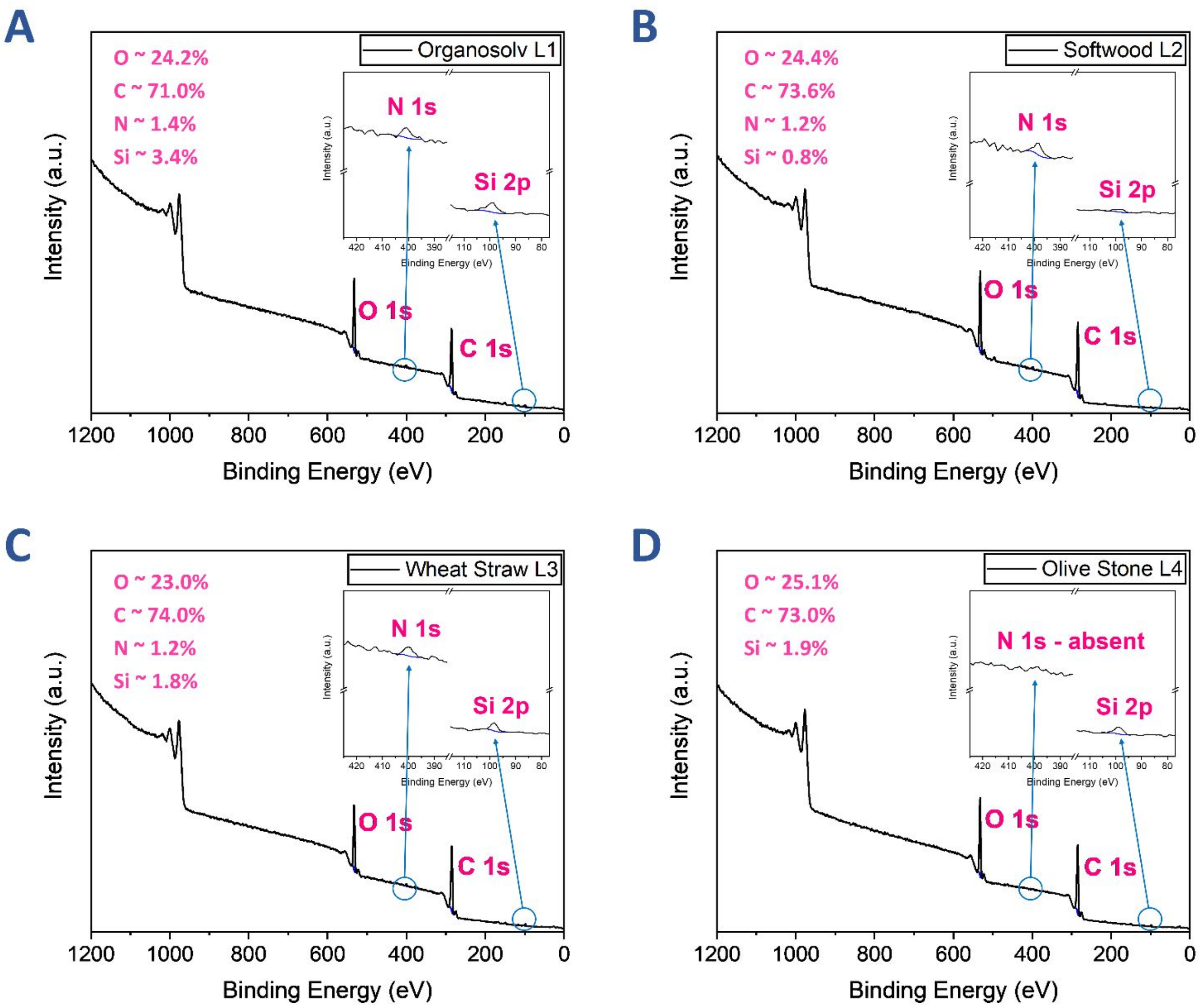


**Figure S11.** Survey XPS spectra of nickel oxide membrane on native oxide silicon wafer **A.** after UVO treatment, **B.** after calcination. XPS high resolution spectra of nickel oxide membrane on native oxide silicon wafer after UVO: **C.** Ni 2p core scan and **D.** O 1s core scan.

## Section S4 – Pattern transfer and optical setup.

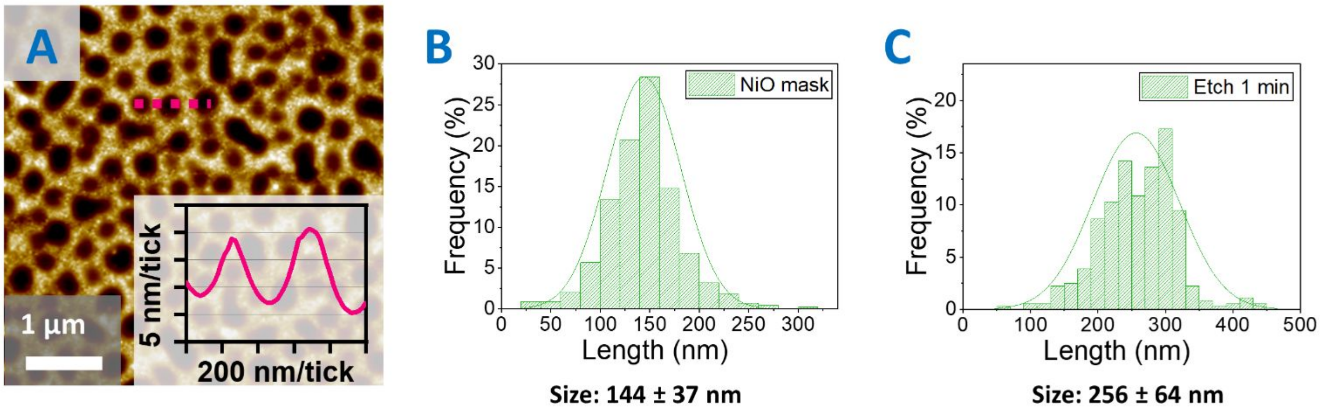


**Figure S12. A.** AFM image of $Ni^{2+}$ bound to lignin **L1** 0.5 wt% membrane on NO silicon substrate after 3 months storage at ambient lab conditions, (no UVO treatment). Pore diameters statistics of **B.** calcined nickel oxide mask and **C.** 1 min etch.

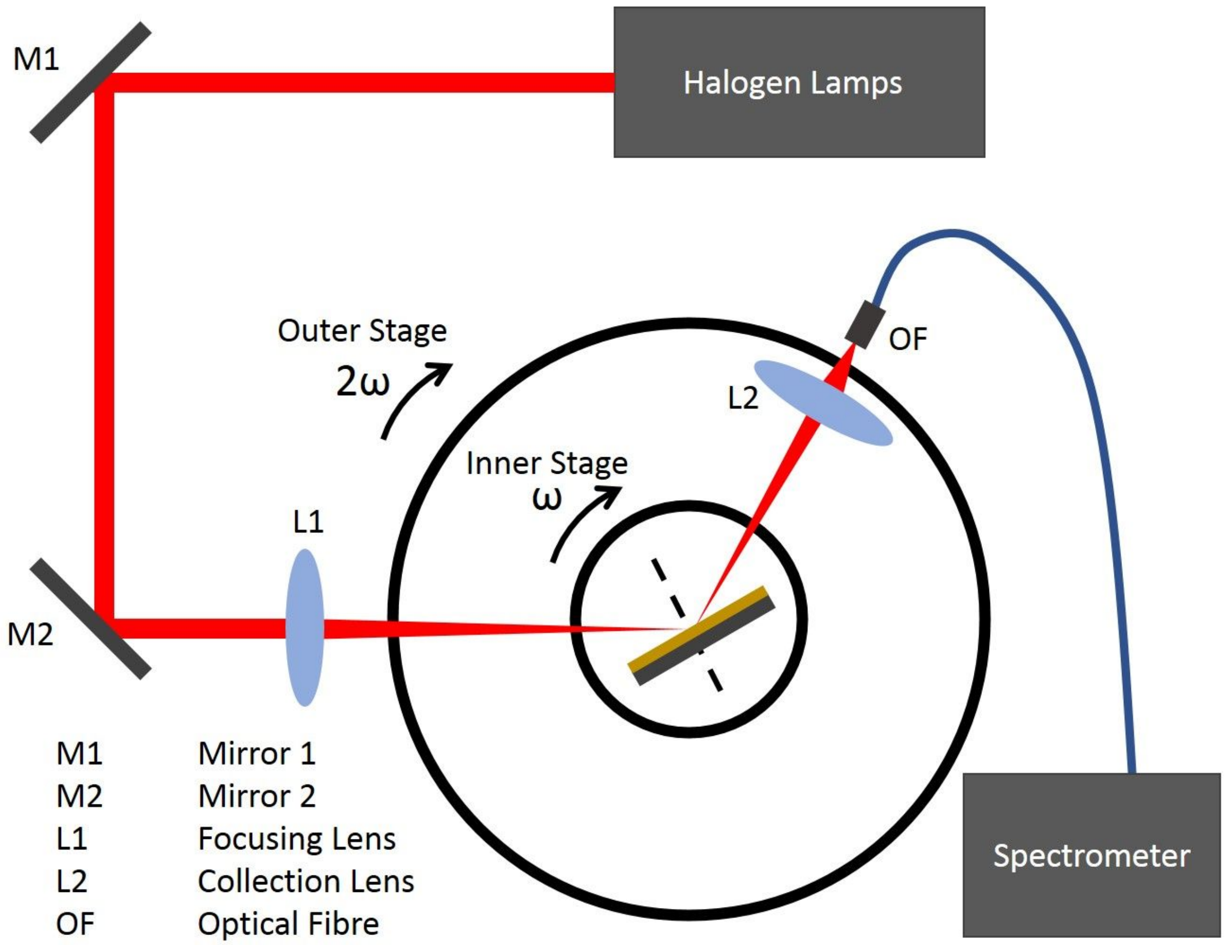


**Figure S13.** Custom built 2Θ system for angle-resolved reflectivity measurements.

# References

(1) Trubetskaya, A.; Souihi, N.; Umeki, K. Categorization of Tars from Fast Pyrolysis of Pure Lignocellulosic Compounds at High Temperature. *Renew. Energy* **2019**, *141*, 751–759. https://doi.org/10.1016/j.renene.2019.04.033.

(2) Trubetskaya, A.; Johnson, R.; Monaghan, R. F. D.; Ramos, A. S.; Brunsvik, A.; Wittgens, B.; Han, Y.; Pisano, I.; Leahy, J. J.; Budarin, V. Combined Analytical Strategies for Chemical and Physical Characterization of Tar from Torrefaction of Olive Stone. *Fuel* **2021**, *291*, 120086. https://doi.org/10.1016/j.fuel.2020.120086.

(3) Trubetskaya, A.; Lange, H.; Wittgens, B.; Brunsvik, A.; Crestini, C.; Rova, U.; Christakopoulos, P.; Leahy, J. J.; Matsakas, L. Structural and Thermal Characterization of Novel Organosolv Lignins from Wood and Herbaceous Sources. *Process. 2020, Vol. 8, Page 860* **2020**, *8* (7), 860. https://doi.org/10.3390/PR8070860.

(4) Fernández-Bolaños, J.; Felizón, B.; Heredia, A.; Guillén, R.; Jiménez, A. Characterization of the Lignin Obtained by Alkaline Delignification and of the Cellulose Residue from Steam-Exploded Olive Stones. *Bioresour. Technol.* **1999**, *68* (2), 121–132. https://doi.org/10.1016/S0960-8524(98)00134-5.

(5) Rashid, T.; Kait, C. F.; Murugesan, T. A "Fourier Transformed Infrared" Compound Study of Lignin Recovered from a Formic Acid Process. *Procedia Eng.* **2016**, *148*, 1312–1319. https://doi.org/10.1016/j.proeng.2016.06.547.

(6) Shi, Z.; Xu, G.; Deng, J.; Dong, M.; Murugadoss, V.; Liu, C.; Shao, Q.; Wu, S.; Guo, Z. Structural Characterization of Lignin from D. Sinicus by FTIR and NMR Techniques. *Green Chem. Lett. Rev.* **2019**, *12* (3), 235–243. https://doi.org/10.1080/17518253.2019.1627428.
